# Isolation of a Novel Swine Influenza Virus from Oklahoma in 2011 Which Is Distantly Related to Human Influenza C Viruses

**DOI:** 10.1371/journal.ppat.1003176

**Published:** 2013-02-07

**Authors:** Ben M. Hause, Mariette Ducatez, Emily A. Collin, Zhiguang Ran, Runxia Liu, Zizhang Sheng, Anibal Armien, Bryan Kaplan, Suvobrata Chakravarty, Adam D. Hoppe, Richard J. Webby, Randy R. Simonson, Feng Li

**Affiliations:** 1 Newport Laboratories, Worthington, Minnesota, United States of America; 2 Department of Veterinary and Biomedical Sciences, South Dakota State University, Brookings, South Dakota, United States of America; 3 Department of Infectious Diseases, St. Jude Children's Research Hospital, Memphis, Tennessee, United States of America; 4 Department of Biology and Microbiology, South Dakota State University, Brookings, South Dakota, United States of America; 5 Department of Chemistry and Biochemistry, South Dakota State University, Brookings, South Dakota, United States of America; 6 Veterinary Diagnostic Laboratory, University of Minnesota, St. Paul, Minnesota, United States of America; Erasmus Medical Center, Netherlands

## Abstract

Of the *Orthomyxoviridae* family of viruses, only influenza A viruses are thought to exist as multiple subtypes and has non-human maintenance hosts. In April 2011, nasal swabs were collected for virus isolation from pigs exhibiting influenza-like illness. Subsequent electron microscopic, biochemical, and genetic studies identified an orthomyxovirus with seven RNA segments exhibiting approximately 50% overall amino acid identity to human influenza C virus. Based on its genetic organizational similarities to influenza C viruses this virus has been provisionally designated C/Oklahoma/1334/2011 (C/OK). Phylogenetic analysis of the predicted viral proteins found that the divergence between C/OK and human influenza C viruses was similar to that observed between influenza A and B viruses. No cross reactivity was observed between C/OK and human influenza C viruses using hemagglutination inhibition (HI) assays. Additionally, screening of pig and human serum samples found that 9.5% and 1.3%, respectively, of individuals had measurable HI antibody titers to C/OK virus. C/OK virus was able to infect both ferrets and pigs and transmit to naive animals by direct contact. Cell culture studies showed that C/OK virus displayed a broader cellular tropism than a human influenza C virus. The observed difference in cellular tropism was further supported by structural analysis showing that hemagglutinin esterase (HE) proteins between two viruses have conserved enzymatic but divergent receptor-binding sites. These results suggest that C/OK virus represents a new subtype of influenza C viruses that currently circulates in pigs that has not been recognized previously. The presence of multiple subtypes of co-circulating influenza C viruses raises the possibility of reassortment and antigenic shift as mechanisms of influenza C virus evolution.

## Author Summary

Influenza C viruses infect most humans during childhood. Unlike influenza A viruses, influenza C viruses exhibit little genetic variability and evolve at a comparably slower rate. Influenza A viruses exist as multiple subtypes and cause disease in numerous mammals. In contrast, influenza C viruses are comprised of a single subtype in its primary human host. Here we characterize a novel swine influenza virus, C/swine/Oklahoma/1334/2011 (C/OK), having only modest genetic similarity to human influenza C viruses. No cross-reaction was observed between C/OK and human influenza C viruses. Antibodies that cross react with C/OK were identified in a significant number of swine but not human sera samples, suggesting that C/OK circulates in pigs. Additionally, we show that C/OK is capable of infecting and transmitting by direct contact in both pigs and ferrets. These results suggest that C/OK represents a new subtype of influenza C viruses. This is significant, as co-circulation of multiple subtypes of influenza allows for rapid viral evolution through antigenic shift, a property previously only shown for influenza A viruses. The ability of C/OK to infect ferrets along with the absence of antibodies to C/OK in humans, suggests that such viruses may become a potential threat to human health.

## Introduction

Influenza A, B and C viruses are members of the *Orthomyxoviridae* family that can cause influenza in humans [1]. Influenza A viruses exist in humans, various other mammal species, and birds; migratory or domestic waterfowl are their largest reservoir. Humans are thought to be the primary hosts and reservoir of influenza B and C viruses, although both have been identified in other hosts after reverse zoonotic transmission from humans. While influenza B virus is a common seasonal human pathogen similar to influenza A virus in its clinical presentation, influenza C virus causes primarily upper respiratory tract infections in children [2]. Clinical manifestations (cough, fever, and malaise) are typically mild, but infants are susceptible to serious lower respiratory tract infections [3]. Influenza C viruses co-circulate with influenza A and B viruses and causes local epidemics [4], [5]. Six genetic and antigenic lineages of influenza C viruses have been described, and as in influenza B viruses, are considered monsubtypic [6], [7]. Co-circulation of multiple subtypes of influenza allows for rapid viral evolution through the process of antigenic shift, a property previously only shown for influenza A viruses. Thus, both influenza B and C viruses do not have pandemic potential. In contrast, the Influenza A genus includes 17 hemagglutinin and 9 neuraminidase subtypes, and reassortment among different subtypes has repeatedly generated pandemic viruses to which the human population is naïve [8]–[10]. It is the animal reservoirs of diverse influenza A viruses that give them the unique property within orthomyxoviruses of causing human pandemics.

Aside from humans, influenza C virus has been isolated only from swine in China (in 1981) [11]. Genetic analysis showed a close relation between Japanese human and Chinese swine influenza C isolates [12], [13]. Serological surveys in Japan and the United Kingdom found 9.9% and 19% of swine, respectively, to have positive HI antibody titers to human influenza C viruses, suggesting that the virus is not uncommon in swine [14], [15]. Swine inoculated with influenza C virus had mild respiratory disease and transmitted the virus to naive swine by direct contact [11]. Here we characterize an orthomyxovirus isolated from a clinically ill pig and show that the virus is distantly related to human influenza C virus and readily infects and is transmissible in both ferrets and pigs. Genetic and antigenic analysis suggest that this virus represents a new subtype of influenza C virus, raising the possibility of reassortment and antigenic shift as mechanisms for influenza C virus evolution which could pose a potential threat to human health.

## Results

### Identification of an Influenza C virus in Pigs

**Figure 1:**
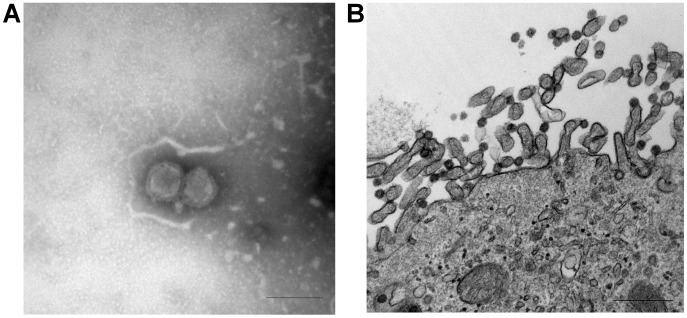
Ultrastructural analysis of C/OK virus isolate in cell culture as observed by negative stain (A) and thin-section (B) electron microscopy. (**A**) Negative stain shows features of an orthomyxovirus particle (Bar = 200 nm) (**B**) Infected cells visualized with uranyl acetate and lead contrast. Note assembly and budding of virions at the apical pole. Free spherical virions 70–90 nm in diameter present surface spikes and internal electron-dense dots. Bar = 500 nm.

In April 2011, nasal swabs from 15-week old swine exhibiting influenza-like illness were submitted to Newport Laboratories, Worthington, Minnesota, for virus isolation. Real-time reverse transcription PCR (rt-RT-PCR) was negative for influenza A virus [16]. In swine testicle (ST) cells, the viruses caused influenza-like cytopathic effects (CPE) by day 3. The cell culture harvests were again negative for influenza A virus by rt-RT-PCR. Electron microscopic (EM) studies of the cell cultures demonstrated features characteristic of an Orthomyxovirus (Fig. 1). Negative-staining EM showed enveloped spherical to pleomorphic viral particles approximately 100–120 nm in diameter (Fig. 1A). The virion surface contained dense projections 10–13 nm in length and 4–6 nm in diameter. Thin-section EM studies of infected cells revealed filamentous budding of virions from the plasma membrane (Fig. 1B). These data strongly suggested the virus to be a member of the family *Orthomyxoviridae*. Enzymatic assays revealed that the virus had negligible neuraminidase but detectable O-acetylesterase activity using 4-nitrophenyl acetate, suggesting it to be a member of the influenza C genus. However, further RT-PCR analysis was negative for influenza B and C viruses [17]. RT-PCR or PCR assays to detect porcine reproductive and respiratory syndrome virus, porcine coronavirus, and porcine circovirus were also negative (data not shown).

### Genome sequencing and analysis

The virus was purified by ultracentrifugation and sequenced on an Ion Torrent Personal Genome Machine. *De novo g*enome assembly found that most of the sequence reads mapped to seven contigs of approximately 1000–2400 bp. Open reading frame (ORF) analysis of the contigs found a single ORF for all segments, with the exception of two ORFs for the smallest contig. BlastP searches of the putative proteins identified modest homology to human influenza C virus, suggesting that this virus was distantly related to human influenza C virus (Table 1). Consequently, the virus was provisionally designated C/swine/Oklahoma/1334/2011 (C/OK). The genomic coding sequences of all segments were determined and used for subsequent genetic and phylogenetic analyses.

**Table 1:** BlastP analysis of the eight putative open reading frames of C/swine/Oklahoma/1334/2011.

ORF (Amino acid)	Best blast hit (virus; accession number)	Identity (%)	Positive (%)^b^
772	PB2 (C/Johannesburg/1/66; Q9IMP3)	53	71
758	PB1 (C/Johannesburg/1/66; AF170575)	72	85
710	P3 (C/Ann Arbor/1/50; NC_006309)	50	66
664	HEF (C/Catalonia/1318/2009; HM748631)	53	69
552	NP (C/Johannesburg/4/67; BAL72794)	39	59
387	P42^a^ (C/Taylor/1233/47; FLCT123347)	38	58
243	NS1 (C/Hiroshima/248/2000; AB099621)	33	48
168	NS2 (C/Sao Paulo/378/82; AB035366)	29	48

a M1 and M2 proteins were not analyzed because the splicing donor and acceptor sites are not predictable due to sequence variation of M segments between C/OK and human influenza C viruses.

b Positive value indicates the degree of similarity between proteins.

Because PB1 is reported to be the most conserved influenza virus protein, it is frequently used to evaluate the evolutionary relationship among influenza viruses [18]. We firstly performed ClustalW alignment of predicted polymerase basic 1 (PB1) amino acid sequences of influenza A, B, and C viruses. C/OK shared approximately 69%–72% mean pairwise identity to influenza C viruses and 39%–41% identity to influenza A and B viruses. Homology between the influenza A and B PB1 proteins was approximately 61% and intrasubtype PB1 proteins of influenza A viruses are extremely conserved reaching up to 90% homology. PB1 protein alignments indicated that C/OK was more closely related to influenza C viruses than to influenza A and B viruses but more distant from individual members of human influenza C type. Pairwise identity between C/OK and influenza C viruses was considerably lower for polymerase basic 2 (PB2) and polymerase 3 (P3) (53% and 50%, respectively). In influenza A and B viruses segment 3 is referred to as polymerase acidic (PA) protein because of its pKa of approximately 5.2. Conversely, segment 3 of influenza C viruses encodes a polymerase with a neutral pH (pKa ∼7.2) and is referred to as P3 [18]. Interestingly, the predicted pKa of C/OK P3 is 6.2, which is between those of the influenza A/B and influenza C viruses.

In influenza C virus, a hemagglutinin esterase (HE) protein is responsible for receptor binding, receptor destroying (acetylesterase), and membrane fusion activities, whereas in influenza A and B viruses, separate hemagglutinin (HA) and neuraminidase (NA) proteins perform these functions in a cooperative fashion. The pairwise sequence identity of the C/OK and human influenza C virus HE proteins was 53%, similar to the 49% observed across the influenza A HA subtypes [10] but clearly higher than the HA homology (approximately 25–30%) between influenza A and B viruses. NS1 of C/OK virus had the lowest homology to its counterpart in human influenza C viruses (29%–33% identity), similar to the less conserved influenza A and B NS1 proteins (22% identity).

Like PB1, the nucleoprotein (NP) and matrix (M) proteins are highly conserved among members of each genus of influenza viruses. Despite the high intragenic homology (>85%), NP and M1 are highly variable among the three influenza virus genera and their intergenic homologies are only about 20–30%, which serve as genus-specific antigens that distinguish between the influenza A, B, and C viruses [19], [20]. The amino acid sequence of the C/OK NP had 38%–41% identity to influenza C viruses. Unspliced mRNA from the C/OK M segment 6 encodes the polyprotein P42, which is cleaved by a signal peptidase to yield M1′ and CM2 [21]. P42 was analyzed due to unknown mRNA splice and protein cleavage sites used by C/OK virus to generate M1 and CM2, respectively. The C/OK P42 had 38% identity to influenza C viruses. Relative low homologies of NP and M proteins between C/OK and human influenza C viruses are interesting but seem to be consistent with pairwise protein homology analysis for polymerase and non-structural proteins.

In addition to the coding region, each RNA segment of influenza viruses also contains noncoding (NC) regions at its 5′ and 3′ ends. These NC regions are highly conserved, particularly those at the terminal ends, among the genome segments of each species. These regions form panhandle structures by partial inverted complementarity between the 5′ and 3′NC regions and play a critical role in genome replication and packaging [22]–[25]. Using 5′ and 3′ RACE coupled with direct PCR sequencing by the Sanger method, we determined the complete sequences of the 3′ and 5′ NC regions of the seven segments of the C/OK virus (Table S1). The 3′ and 5′ NC region sequences of C/OK genome segment were similar to those of human influenza C viruses with the exception of one nucleotide (position 5 from the 3′-terminus) and polymorphism at position 1 of the 3′terminus.

Viral RNA packaging sequences are composed of the 5′ and 3′NC regions and the terminal coding sequences of each segment. Incompatibility between homologous segment packaging sequences has been shown to prevent segment reassortment [26]. Nearly identical NC sequences at the proximal ends of seven RNA segments observed between C/OK and human influenza C viruses suggest a potential for viral segment reassortment in nature. Significant variability was observed in the NC regions immediately adjacent to each coding region for C/OK as compared to human influenza C; however previous work demonstrated that the highly conserved NC region at the proximal ends of the segment plays a key role in transcription and replication [27].

### Phylogenetic analysis

**Figure 2:**
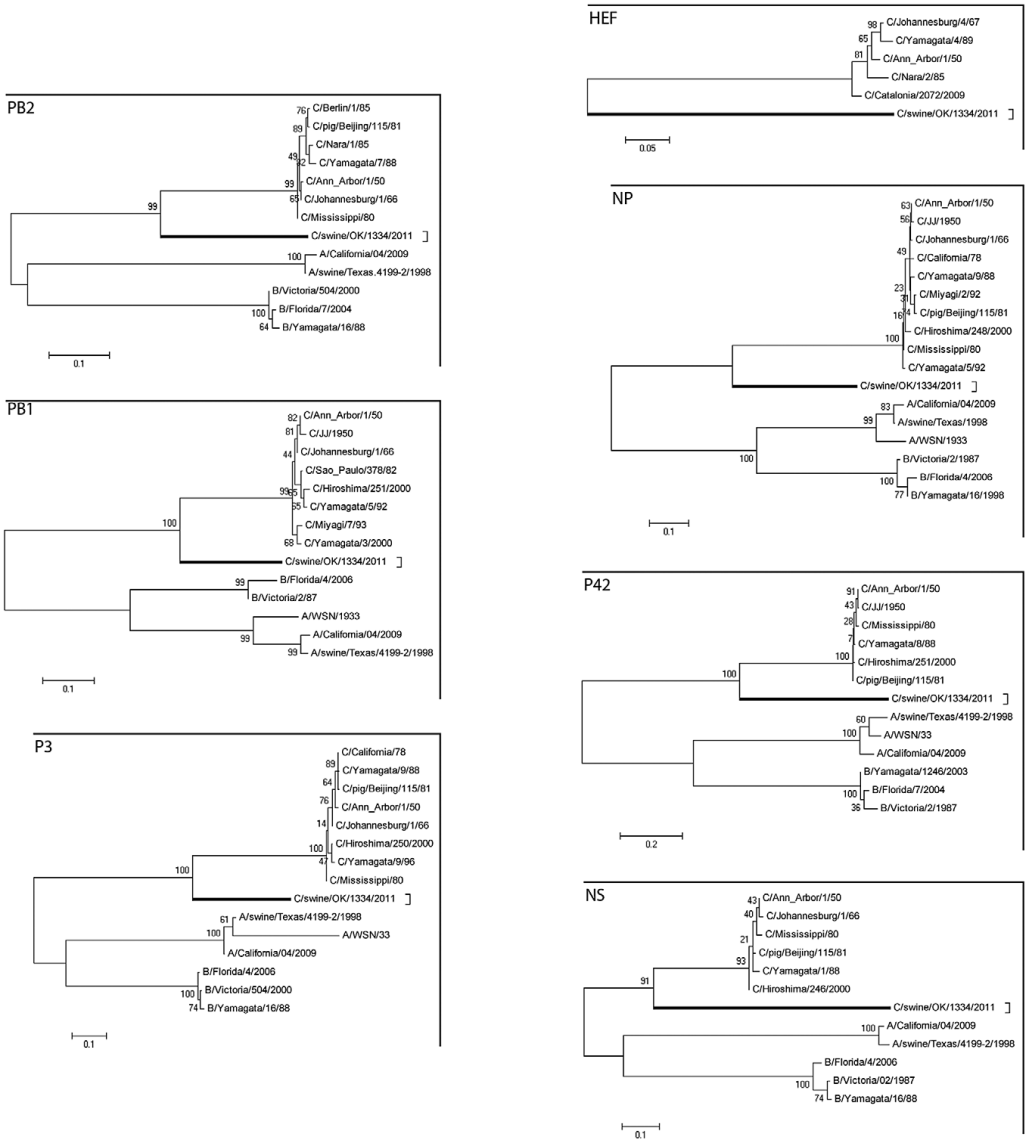
Phylogenetic trees of the coding regions of the seven segments of C/OK virus. Maximum-likelihood analysis in combination with 1000 bootstrap replicates was used to derive trees based on the nucleotide sequences encoding respective proteins. A scale representing the number of nucleotide changes is shown in each panel. The trees are shown for PB2, PB1, P3, HE, NP, P42, and NS segments. Bootstrap values are shown above branches to the left of major nodes.

Our phylogenetic analysis used representative influenza A, B, and C viruses (Fig. 2). The segments encoding the C/OK virus PB2, PB1, P3, NP, M and NS clustered most closely with influenza C viruses, suggesting that these C/OK genes diverged from known human influenza C viruses after they diverged from influenza A and B viruses but before they diverged from previously sequenced influenza C viruses. As HE does not occur in influenza A and B viruses, only influenza C viruses were included in that analysis. Previous studies have found that multiple genetically and antigenically distinct but related lineages of influenza C virus co-circulate and frequently reassort [6], [28]–[30]. Given this evidence, it is puzzling that the seven segments of C/OK are only slightly to moderately homologous to characterized influenza C viruses.

### Serological survey

An HI assay was performed to determine the antigenic cross-reactivity and seroprevalence of C/OK virus in humans and swine. The assay included reference strains of influenza A, B, and C genera and their matched antisera (Table S2). No cross-reactivity was observed between C/OK virus and heterologous antisera. For the human cohort, we used a set of 316 serum samples.

These sera originated from patients recruited in the Greater Vancouver area of British Columbia, Canada, or in the vicinity of the Greater Hartford area of Connecticut during the 2007–2008 and 2008–2009 influenza seasons as described in Marcelin et al. [31]. All but four of these sera had undetectable C/OK HI titers (≤10). Three had HI titers of 20, but each of these also had high titers (160, 320, and 1280) to the human influenza C isolate C/Yamagata/10/1981. The remaining positive sample had a HI titer to C/OK of 40 with no corresponding titer to the human influenza C isolates tested. The low titers and number of positive samples (1.3%) obtained are inconclusive in determining circulation of C/OK in the human population, especially as thirty-four percent of the serum samples had HI titers ≥20 to C/Yamagata/10/1981 which is consistent with previous studies showing approximately 60% of elderly humans retain influenza C virus antibody titers [32]. Swine serum samples (*n* = 220) submitted to Newport Laboratories for unrelated diagnostic testing by commercial swine production facilities nationwide were similarly analyzed. Sera were collected from pigs aged 3–20 weeks from March through September 2011. HI titers (range, 10–80) were detected in 9.5% of samples, with a GMT of 20.7. To assess the specificity of the HI titers to C/OK in swine sera, we performed HI assays using the human influenza C virus C/Taylor/1233/47 (C/Taylor). Only 2.8% of the swine sera had measurable titers (range, 10–20). Taken together, these results suggest that C/OK virus circulates in swine populations but is not widespread in humans. Further serologic studies focusing on individuals occupationally exposed to swine are required.

### Infection and transmission in ferrets and pigs

**Figure 3:**
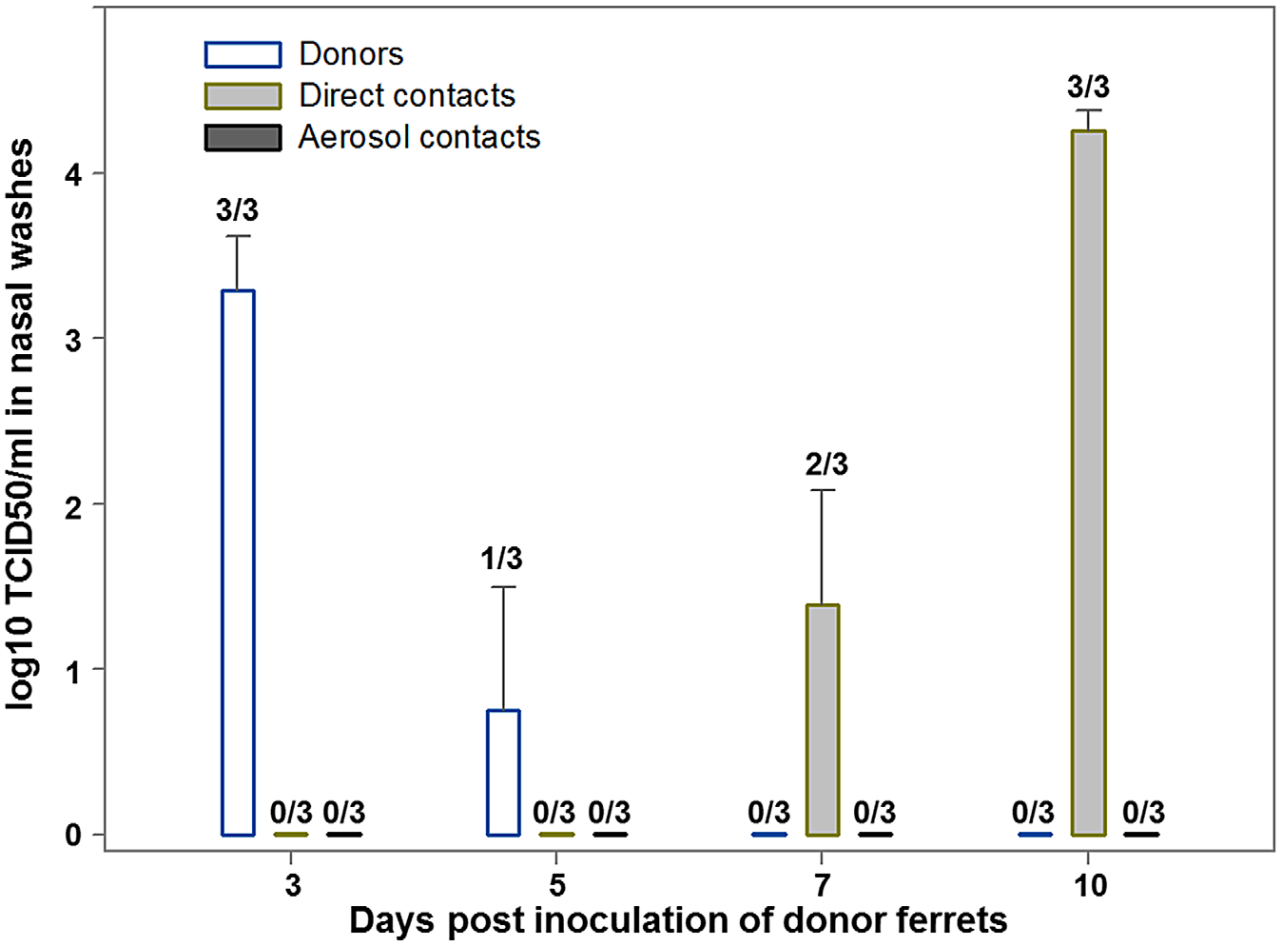
Replication of C/OK virus in ferrets challenged intranasally (donors) or exposed by direct or aerosol contact. Three ferrets (donor ferrets) were inoculated intranasally with 10^6^ TCID_50_ of C/OK virus in 1 ml of sterile PBS. Two inoculated ferrets were also housed separately for virus titration and histopathology in organs. At 23 h p.i., each of the three remaining donor ferrets was housed in a cage with one naïve direct-contact ferret (n = 3). An additional ferret (n = 3) was placed in an adjacent cage separated from the donor's cage by a two layers of wire mesh (∼5 cm apart) that prevented physical contact but allowed the passage of respiratory droplets. To monitor virus shedding, nasal washes were collected from ferrets 3, 5, 7, and 10 days p.i. Virus was titrated in ST cells as log_10_ TCID_50_/ml and values shown are the mean ± standard error.

To better understand the pathogenesis and epidemiology of C/OK, we performed infection studies with ferrets and swine. We first addressed the zoonotic potential of C/OK virus by conducting a pathogenesis and transmission study in the ferret model. After intranasal inoculation of ferrets, C/OK virus was first detected in nasal washes on day 3 (mean titer, 3.3 log_10_ TCID_50_/mL) (Fig. 3). C/OK virus was first detected in ferrets exposed by direct contact to inoculated ferrets on day 7, reaching a mean titer of 4.3 log_10_ TCID_50_/mL by day 10. Virus was not detected in ferrets exposed to respiratory droplets. No clinical signs of disease were observed. In the tissues of ferrets on day 5 post-inoculation (p.i.), a mean titer of 3.9 log_10_ TCID_50_/mL was observed in the nasal turbinates, but no virus was detected in the upper and lower trachea, lung, small intestine, liver, or spleen. Histopathological examination of lung tissues showed no typical influenza lesions. These results are consistent with a previous study that investigated human influenza C replication in ferret alveolar macrophage cells where viral replication with titers >10^4^ egg infectious dose 50 from days 4 to 9 were measured with no cytopathic effects [33]. All ferrets that were inoculated or exposed by direct contact and 1/3 of the ferrets exposed to respiratory droplets seroconverted 3 weeks after exposure as measured by HI assay (GMT = 780). To assess the pathogenicity and transmissibility of the virus in swine, we similarly challenged swine intranasally with C/OK (Fig. S1). Virus was first detected in nasal swabs on day 3 p.i. by using an rt-RT-PCR method specifically developed for C/OK virus. Virus shedding peaked at day 8 p.i. and remained detectable on day 10. Virus was detected in swine exposed by direct contact on days 7 and 9 after exposure. No clinical signs of illness were observed. Lung samples collected from inoculated swine on day 7 p.i. showed no evidence of the virus by rt-RT-PCR. Histopathological examination of lung tissues showed no typical influenza lesions. Sera collected on day 14 p.i. from donor pigs were positive for antibodies to C/OK virus in an HI assay (GMT = 30.3). All 5 pigs were positive for antibodies to C/OK. Additionally, 2 of the 5 direct contact pigs seroconverted by day 13 post exposure. These data suggest that in animals the replication kinetics is slower for C/OK virus than for influenza A viruses and infection in both swine and ferrets was limited to the upper respiratory tract. The ability of C/OK to readily transmit to contact ferrets suggests that a level of transmission potential to humans is possible. Zoonotic H5N1 and H9N2 influenza A viruses are typically unable to transmit in ferrets.

### Cellular tropism

We compared *in vitro* cellular tropism between C/OK and human influenza C viruses by rt-RT-PCR in several cell lines, including ST, adenocarcinomic human alveolar basal epithelial (A549), Madin-Darby canine kidney (MDCK), Green African monkey kidney (Marc-145), human rectal tumor (HRT-18G), baby hamster kidney (BHK-21) and porcine kidney (PK-15) cells. C/OK replicated, in order of highest replication, in ST, MDCK, Marc-145, HRT-18G and A549 cells (Fig. S2A). Minimal replication was observed in BHK-21 and PK-15 cells. In marked contrast, the human influenza C virus C/Taylor showed poor growth and replicated only in ST and HRT-18G and not in other cells tested (Fig. S2B). It should be noted however that cultivation of influenza C/Taylor virus was performed at 33°C, the optimal temperature that is typically used to propagate human influenza C virus [34]. This virus failed to replicate at 37°C in our hands which differs from the C/OK virus that can replicate efficiently at this temperature (data not shown). These results suggest that C/OK has a broader cellular tropism than C/Taylor and is also not restricted at elevated temperatures for replication.

### Structural basis for the observed difference in cellular tropism

**Figure 4:**
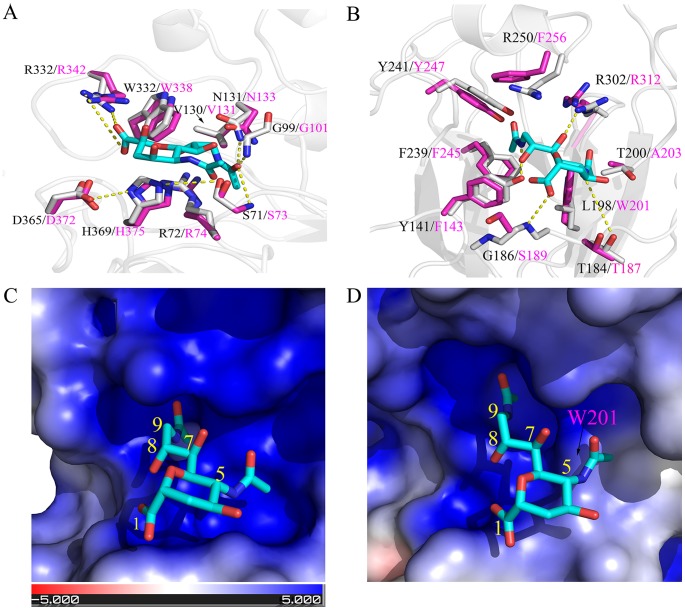
Structural comparisons of C/OK and human C HE receptor-binding pocket and substrate-binding site. (A) Superposition of the esterase active site of C/OK (magenta sticks) and human C/Johannesburg/1/66 (gray sticks). The potential hydrogen bonds shown were from a previous study and were indicated as dashed line [36]. The analog of 9-O-sialic acid, 9-acetamindo-sialic acid α-methylglycoside, was colored cyan. (B) Superposition of the sialic acid binding site of the receptor binding domain of C/OK (magenta sticks) and human C/Johannesburg/1/66 (gray sticks). Hydrogen bonds between residues of C/Johannesburg/1/66 and sialic acid were shown as dashed yellow line. The structure of C/Johannesburg/1/66 HEF was transparently shown in cartoon mode (gray). (C) Electrostatic surface of human C/Johannesburg/1/66 HE. Blue colored region has strong positive potential while red colored region has negative potential. (D) Electrostatic surface of C/OK HE. For both (C) and (D), the nine carbons of sialic acid are numbered.

The influenza C virus utilizes the 9-*O-*acetyl-*N*-acetylneuraminic acid (Neu5,9Ac2) as the primary receptor for attachment to the cell surface to initiate infection [35]. The receptor binding specificity and affinity are mainly determined by the 9-O-acetyl group of the Neu5,9Ac2 [36]. As a result, the virus encodes a sialate-*O*-acetylesterase, not neuraminidase, in order to release virions from infected cells by cleavage of the 9-O-acetyl group [37]. To provide structural insights to the observed different tropism, we conducted structural modeling of the C/OK HE protein in complex with the receptor based on the solved X-ray crystallographic structure of a human influenza C virus (C/Johannesburg/1/66) HE protein [36]. The overall 53% sequence identity between the two HE proteins allows us to predict important structural features such as the receptor-binding pocket and the enzymatic active site of the C/OK HE protein. Based on the assumption that the HE protein uses similar sites for function, our structural modeling analysis identified a conserved enzymatic active site but revealed a variable receptor-binding pocket between two HE proteins (Figs. 4 and S3). These results are consistent with the observed difference in cellular tropism. For example, both HE proteins possess an identical catalytic triad: S71/H369/D365 for C/Johannesburg and S73/H375/D372 for C/OK (Fig. 4A). The other two substrate-interacting residues for optimal enzymatic function are also completely conserved in the two HE proteins (G99/N131 for C/Johannesburg and G101/N133 for C/OK). In addition, both HE proteins utilize two conserved arginine residues for substrate binding (R72/R332 for C/Johannesburg and R74/R342 for C/OK). The conserved enzymatic site between C/Johannesburg and C/OK suggests that C/OK utilizes 9-O-acetyl sialic acid as the cellular receptor for infection.

Analysis of the receptor-binding pocket formed by a cluster of noncontiguous amino acid residues revealed some interesting similarities and differences between C/Johannesburg and C/OK. The influenza C HE protein uses two binding pockets for recognizing the receptor: one binds to the 9-*O-*acetyl group while the other engages the 5-*N-*acetyl group [36]. As shown in Fig. 4, the 5-*N-*acetyl binding pocket of C/OK HE becomes smaller as compared to that of C/Johannesburg due to L→W substitution, i.e. L198 in C/Johannesburg is replaced by W201 at C/OK HE position 201; the large rigid aromatic side-chain of W201 extends into the binding pocket (Fig. 4B, 4C, and 4D). The binding pocket for the 9-*O-*acetyl group is nearly identical in C/Johannesburg and C/OK, implying the utility of the 9-*O-*acetyl-neuraminic acid for C/OK virus infection. For interacting with the 9-*O*-acetyl group, human influenza C viruses utilize a cluster of amino acid residues Y141, F239, Y241, R250, and R302, which is also used by the C/OK virus except for two phenylalanine residues replacing tyrosine (Y141) and arginine (R250), respectively. We hypothesize that these amino acid differences may alter the binding specificity and affinity of the HE protein to the receptor that in turn result in the observed difference in cellular tropism between two viruses.

## Discussion

Previous studies have indicated the presence of multiple lineages and antigenic groups in influenza virus type C virus [28]–[30]. Despite such variation, it has been long viewed that the influenza C viruses consist of a single subtype [1]. In contrast to this conventional wisdom, here we describe the characterization of a novel influenza C virus from swine with influenza-like illness. The phylogenetic analyses, together with the observations of genomic structure, indicated that this novel virus is more closely related to influenza C than to other members of the *Orthomyxoviridae* family including influenza A and B viruses, and suggested that the virus could be considered a new subtype of influenza C virus, despite its divergence from human influenza C viruses, which is similar to the divergence between influenza A and B viruses. Identification of this virus was not an isolated case, as we have identified four additional swab samples from swine showing influenza-like symptoms that were positive for this virus in a RT-PCR assay (data not shown). These samples were collected from different pig farms across the U.S. between 2010 and 2012.

The finding that influenza C virus, like influenza A, harbors multiple subtypes is significant. It suggests the possibility of reassortment between subtypes, which could potentially generate viruses with phenotypes that may pose a threat to public health. Nine and a half percent of surveyed swine possessed antibodies specific to C/OK indicating that this previously unidentified virus circulates in U.S. swine. A causal relationship is evidently supported by the experimental infection of pigs. As such, reassortment may occur between C/OK and human influenza C viruses in pigs because both viruses can infect and transmit among pigs and because swine has been documented in serving as a mixing vessel for reassortment of influenza A viruses [38], [39].

Despite a lack of compelling evidence of C/OK virus infection in humans, we suspect that the virus may infect and replicate in the human population because of the following reasons: First of all, the virus infects and transmits in ferrets, a surrogate for human influenza pathogenesis studies. The ability of C/OK virus to readily transmit to contact ferrets suggests that a level of transmission potential to humans is possible. Second, C/OK virus displays a broader cellular tropism compared to human influenza C virus, and this virus seems quite plastic in terms of propagation because high temperatures such as 37°C do not restrict its replication at least in cell culture. Human influenza C virus causes a mild respiratory disease in humans and the infection is normally confined to the upper respiratory tract, although occasionally it can also cause lower respiratory infection [5]. Considering that C/OK virus varies significantly from currently circulating human influenza C viruses in the amino acid sequences of predicted proteins, assessment of clinical disease in humans, particularly in children, caused potentially by C/OK is justified. Knowledge of its presence in human clinical settings is important to any future attempt to manage and control the disease outbreak. By using the nucleotide sequence of C/OK virus reported in this work, sensitive and specific diagnostic methods can be developed to investigate the pathogenesis and epidemiology of this novel virus in humans.

Influenza C virus is not readily isolated and cultured, and primary isolation can be challenged. The obstacle is largely due to a lack of suitable cell lines for influenza C virus isolation as suggested previously [5], [34]. In contrast, identification and cultivation of the C/OK in the ST cell line is relatively straightforward. The emerging but puzzling question then is why this virus has not been identified until now. We suspect that several factors including use of less susceptible cell lines and complicated co-infection often involving influenza A viruses and other viruses having the capacity to agglutinate red blood cells may account for the previous failures in identifying this virus. Alternatively, the C/OK virus may have spread to swine in recent years from an unknown animal reservoir. On-going retrospective seroepidemiological analyses will help to address this question.

The difference in cellular tropism between C/OK and human influenza C may be a result of differences in the receptor recognition of the HE protein. To explore the possible structural origins of this difference, we created a homology model for C/OK using the crystal structure of the influenza C HE protein (Fig. S3). The high sequence identity (53%, Fig. S3) between C/OK HE and influenza C suggest that the quality of this model will be high, given previous work in homology modeling and structure validation for influenza A and B HA proteins in which similar sequence similarities gave RMSD errors of ∼1 Å [40], [41]. The predicted receptor binding for C/OK HE appears on the top face of the receptor binding domain similar to human influenza C HE protein and remote homolog HE proteins of other viruses such as coronavirus and torovirus (∼30% sequence similarity to influenza C HE) [42]. Here, four out of nine residues of the receptor binding site and residues around the binding pocket of HE of human influenza C are retained in that of C/OK (Fig. S4). Previous work has shown that receptor binding specificity and affinity are sensitive to substitutions in the receptor binding site of HE and HA [43], [44]. Examining the predicted receptor-binding site of C/OK HE revealed changes in the receptor binding site relative to human influenza C HE. The most notable difference is a reduction in the size of the 5-*N-*acetyl binding pocket of C/OK HE relative to C HE due to the L198 in C HE being replaced by W in C/OK HE (Fig. 4). Alternatively, the binding pocket for the 9-*O-*acetyl group is similar between the two HE proteins (Fig. 4). We speculate that these differences may indicate that C/OK HE utilizes a different substrate than influenza C HE. Further experimental verifications of are required to test this prediction.

It has been suggested that influenza A, B, C viruses have a common precursor, and of the three virus types, influenza A and B viruses are much more similar to each other in genome organization and protein homology than to C viruses, which suggests that influenza C virus diverged well before the split between A and B viruses [9]. Numerous studies have shown that influenza C viruses have the slowest evolutionary rate among influenza viruses [13], [19], [45]–[47]. One theory is that influenza C viruses, like influenza B viruses, are close or at an evolutionary equilibrium in humans, whereas influenza A viruses have not yet reached an equilibrium [48]. Consistent with this hypothesis is that only a single subtype is thought to exist for influenza B and C viruses and humans, not other mammals, are the primary hosts of influenza B and C viruses. The discovery of C/OK in pigs, being distantly related to human C viruses, seems to challenge these accepted views and warrant future studies of influenza C virus evolution.

Virus nomenclature is the subject of discussion and there is still a possibility that C/OK virus can be assigned as the prototype of a new genus of the *Orthomyxoviridae* family. Most compelling evidence in support of the tentative designation of the C/OK virus are (i) seven genomic segments, (ii) 3′ and 5's NC regions similar to those of human influenza C viruses, and (iii) HE protein sharing approximately 53% homology with that of influenza C viruses. The last parameter is the primary determinant to classify subtypes of influenza A virus. However, the overall divergence between C/OK and human influenza C viruses is similar to that observed between influenza A and B viruses and argues for classification of C/OK into a potential new virus genus. Influenza A, B, and C viruses are classified on the basis of antigenic differences between their nucleoprotein (NP) and matrix (M) proteins [1]. Intriguingly, only modest homologies of these structural proteins between C/OK and human influenza C further confound the provisional classification for this newly discovered virus. Recently the family Orthomyxoviridae was expanded by including two novel genera, Thogotovirus, consisting of three viruses that infect birds and ticks, as well as the genus Isavirus, consisting of infectious salmon anemia virus [49], [50]. For the novel swine influenza virus reported here, perhaps not until attaining more detailed serological, virological, and molecular data, a final classification of this virus can be made. Of particular importance are reassortment experiments between C/OK and human influenza C viruses. While similar, two discrepancies were found in the NCR's of C/OK as compared to human influenza C viruses. It is not known whether these mutations prevent reassortment between C/OK and human influenza C viruses. Preliminary reassortment experiments between C/OK and C/Taylor have been performed and have failed to identify reassortant viruses. More detailed studies are underway.

In summary, we identified a novel influenza C virus that infects and spread among pigs or ferrets by direct contact. The ability of this novel pathogen to infect ferrets; a surrogate for human influenza infection suggests that such viruses may become a potential threat to human health. Our finding reported in the present study raises several interesting questions. Does this influenza C-like virus have the capability of generating a viable reassortant with currently circulating human C viruses? If so, could such a reassortment allow influenza C virus to diverge and to have greater pathogenicity? When and where did this novel virus emerge? What is its animal reservoir in nature? Future elucidation of these questions will provide insights into the ecology, virology, and pathobiology of influenza C virus.

## Materials and Methods

### Ethics statement

Ferret experiments were conducted in an Animal Biosafety Level 2+ (level 2 with enhanced biocontainment for pandemic H1N1 influenza A virus) facility at St. Jude Children's Research Hospital, in compliance with the policies of the National Institutes of Health and the Animal Welfare Act and with the approval of the St. Jude Children's Research Hospital Animal Care and Use Committee (IACUC No. 428). Pig experiments were performed at Newport Laboratories under biosafety level 2 conditions in accordance with the Guide for the Care and Use of Agricultural Animals in Research and Teaching and were approved by the Institutional Animal Care and Use Committees at Newport Laboratories (IACUC No. 02-2012).

### Virus isolation

Nasal swabs were collected from 15-week-old pigs exhibiting influenza-like illness at a commercial swine production facility in Oklahoma, USA, in April, 2011. Viral isolation was performed on swine testicle cells and cytopathic effects were evident by day 3 post inoculation. Detailed information on cell culture conditions is available in SI Appendix, Supplementary Materials and Methods. Hemagglutination assays were performed using chicken red blood cells.

### Genome sequencing and analysis

C/OK was concentrated from ST cell supernatants by ultracentrifugation and subsequently purified through a 20% sucrose cushion by ultracentrifugation. Viral RNA was isolated using the Qiagen Viral RNA Isolation Kit and converted to cDNA using random primers included in the GoScript Reverse Transcription Kit (Promega). The cDNA was made double stranded with DNA polymerase and used to construct a library for Ion Torrent Sequencing. Detailed sequencing methodology is available in SI Appendix, Supplementary Materials and Methods.

Contigs were assembled *de novo* by using SeqMan NGen software (DNAStar). Contigs encoding proteins with homology to influenza C proteins were identified by BlastP analysis. The genome sequence of C/OK was submitted to Genbank under accession no. JQ922305-JQ922311, relating to segments 1–7, respectively. Phylogenetic analyses were performed by using Mega 5 software [51]. Evolutionary analyses were conducted by using the Maximum Likelihood algorithm, and the tree topology was verified by performing 1000 bootstrap replicates.

### Real Time Reverse Transcription PCR Assay

The PB1 sequence was used to design primers and a Taqman probe for detection of C/OK (position of primers and probe in PB1 gene: forward, nucleotides 1420–1439; reverse, nucleotides 1555–1535; probe, 1482–1460). Viral RNA was extracted by using the MagMAX-96 viral RNA isolation kit (Life Technologies) according to the manufacturer's instructions. rt-RT-PCR was performed by using QIAGEN Quantitect RT-PCR with the C/OK primers and probe. Method specificity was assessed by using influenza A, B, and C reference viruses, and no cross-reaction was observed.

### Cell culture and virus replication

Swine testicle (ST) cells were grown in DMEM containing 5% fetal bovine serum. Influenza C/Taylor/1233/47 virus was provided by BEI Resources (NIAID). For infection, cell medium was replaced with DMEM and viral inoculum was added at a multiplicity of infection of 0.001. Viral growth studies were performed on a monolayer of ST, A549, Marc145, HRT-18G, BHK-1, or PK-15 cells using an inoculum of 1.0–3.0 TCID_50_/ml in duplicate (multiplicity of infection 1×10^−5^–1×10^−3^). C/OK virus replication was performed at 37°C while C/Taylor replication was done at 33°C. Samples were removed at 0, 24, 48, and 72 hours p.i. and virus was titrated by rt-RT-PCR. Experiments were performed three times in duplicate.

### Serology

Human sera were treated with receptor-destroying enzyme (Denka Seiken Co., Tokyo, Japan) overnight at 37°C, heat-inactivated at 56°C for 30 min, diluted 1∶10 with PBS, and tested by hemagglutination inhibition (HI) assay with 0.5% packed chicken red blood cells (cRBCs) as described in the WHO Manual on Animal Influenza Diagnosis and Surveillance [52].

### Assessment of virus pathogenicity and transmission in ferrets

The pathogenicity and transmission of the virus was tested in 3- to 4 month-old male ferrets. Detailed pathogenicity and transmission methodology is available in SI Appendix, Supplementary Materials and Methods. Three donors were inoculated intranasally under light isoflurane anesthesia with 10^6^ TCID_50_ of swine/Oklahoma/1334/2011 virus in 1 ml of sterile PBS. Two additional ferrets were similarly inoculated and were housed separately for virus titration and histopathology in organs. At 23 h p.i., each of the three remaining donor ferrets was housed in a cage with one naïve direct-contact ferret (n = 3). An additional ferret (n = 3) was placed in an adjacent cage separated from the donor's cage by a two layers of wire mesh (∼5 cm apart) that prevented physical contact but allowed the passage of respiratory droplets. Clinical signs of infection, relative inactivity index [53], weight, and temperature were recorded on days 0, 3, 5, 7, and 10 p.i.. Nasal washes were collected from ferrets 3, 5, 7, and 10 days p.i. Two donor animals were euthanized 5 dpi, and tissue samples were collected. Samples were homogenized and virus was titrated (log_10_ TCID_50_ per gram of tissue) in ST cells. Tissues were also subjected to histopathologic analysis.

### Assessment of virus pathogenicity and transmission in swine

Swine challenge studies were performed at Newport Laboratories under biosafety level 2 conditions. Twenty-eight swine approximately 10 weeks of age were obtained from a commercial high-health herd. Eleven swine were placed in a single room and inoculated intranasally with 6.0 log_10_ TCID_50_ of C/OK. On day 1 p.i., 11 naïve direct-contact swine were introduced into the room. Temperatures were recorded and nasal swabs were collected on days 0, 2, 3, 6, 8, and 10 p.i. Six inoculated swine and three mock-inoculated swine were euthanized on day 7 p.i. and lung specimens were fixed in 10% neutral buffered formalin and submitted for histopathological analysis. The remaining swine were euthanized on day 14 p.i. Nasal swabs and lung tissue were analyzed by rt-RT-PCR as described above.

### Structural modeling

The structure of C/OK HE (aa 17–620) was modeled using Modeller 9.10 [54]. The structure of C/Johannesburg/1/66 HE was used as template (PDB id: 1FLC) [36] because the two HE proteins show both high sequence and secondary structure similarity. The quality of the modeled structure was estimated by Verify_3D and 95.47% of the modeled residues are compatible with the structure (averaged 3D-1D score >0.2) [55], suggesting that the quality of the modeling is good. The distance between the modeled structure and template is 0.49 angstrom. Electrostatic surface maps were generated using APBS [56].

## Supporting Information

Figure S1 **rt-RT-PCR Ct values of nasal swabs from swine inoculated intranasally with C/OK (donors) and from swine exposed to inoculated swine by direct contact.** Numbers of pigs positive by rt-RT-PCR are indicated above or below each time point.
(TIFF)

Figure S2 **Growth of influenza C/swine/Oklahoma/1334/2011 (A) and influenza C/Taylor/1233/1947 (B) in cell cultures.** ST, MDCK, Marc145, PK or -15, BHK-21, HRT-18G and A549 cell lines were inoculated with approximately 1.0–3.0 log_10_ TCID_50_/mL of influenza C/OK virus or influenza C/Taylor virus (MOI = 1×10^−5^–1×10^−3^). Virus was titrated at the indicated time points by rt-RT-PCR. Experiments were done in duplicate; values are the mean of duplicate samples and error bars represent the standard deviation.
(TIF)

Figure S3 **Modeled structure of C/OK HE protein.** Cartoon and surface representations of HE structure are shown below. HE was colored blue. Residues that are not identical in C/Johannesburg/1/66 HE were marked red. An analog of 9-O-sialic acid, 9-acetamindo-sialic acid α-methylglycoside (cyan), was manually docked to the binding sites of receptor and esterase domain according to a previous study [36].
(TIFF)

Figure S4 **Sequence alignment and secondary structure of HE protein.** Sequences were aligned using MUSCLE [57]. Esterase active site residues and receptor binding site residues of human influenza C HE protein are marked with red and blue rectangles, respectively. Secondary structure of C/OK HE was predicted using PSIpred while that of human influenza C HE protein is from PDB structure (1FLC) [58]. Pink rectangles represent α helix, orange arrows represent β strands and black lines are random coils and loops.
(TIF)

Table S1 **Sequences of the 3′ and 5′ noncoding regions of the genomic segments of C/swine/Oklahoma/1334/2011 (A) and C/JHB/1/66 (B).**
(DOCX)

Table S2 **Cross-reactivity of antibodies to influenza A, B and C viruses and C/swine/Oklahoma/1334/2011 virus as measured by HI assay using turkey red blood cells.**
(DOCX)

Text S1 **Supplementary Materials and Methods.**
(DOC)
