# Supplementary figures and images for: Isolation of a Novel Swine Influenza Virus from Oklahoma in 2011 Which Is Distantly Related to Human Influenza C Viruses
Source: PLoS Pathog. 2013 Feb 7;9(2):e1003176. doi: 10.1371/journal.ppat.1003176 (PMC3567177; doi:10.1371/journal.ppat.1003176)

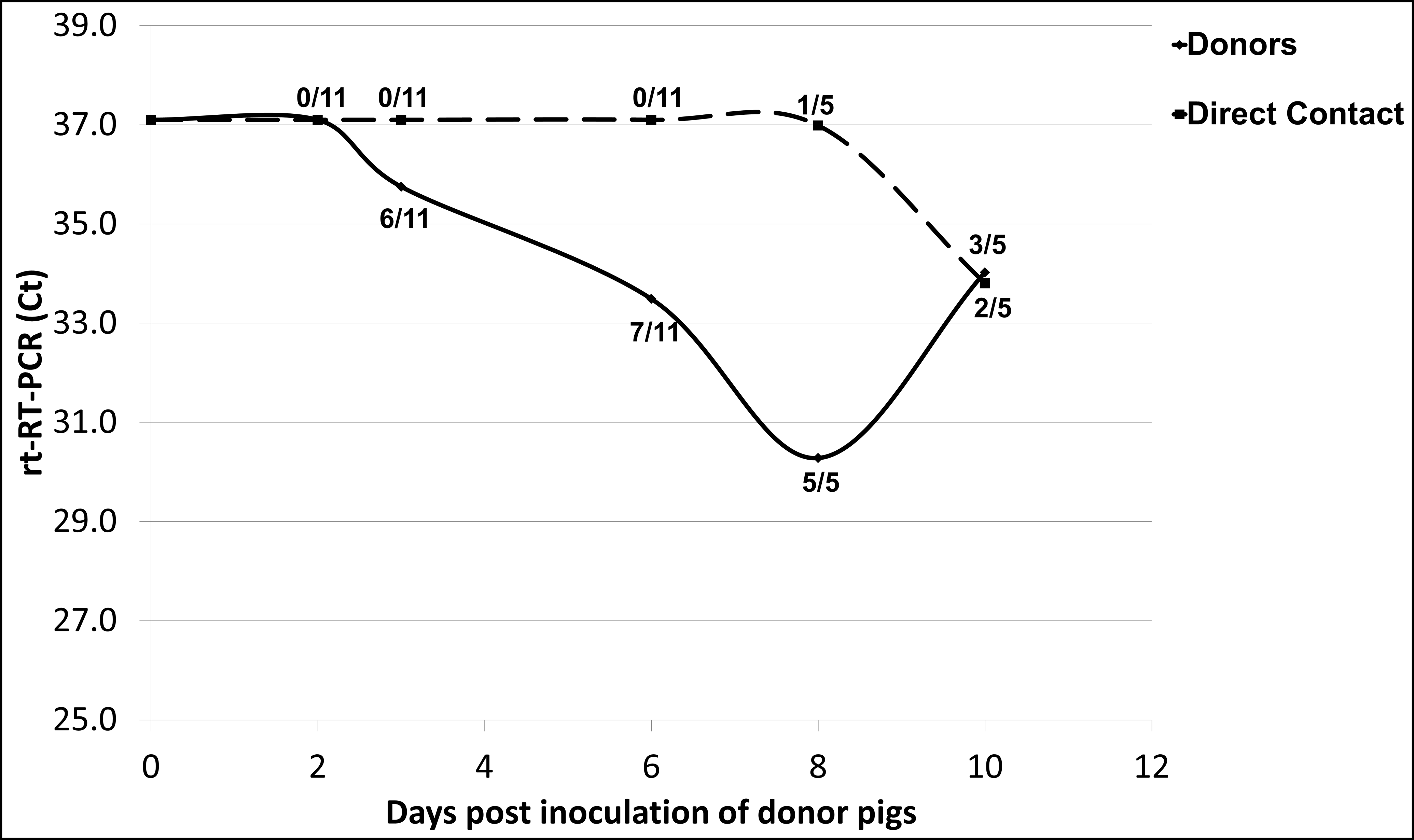

Supplement: Figure S1 — rt-RT-PCR Ct values of nasal swabs from swine inoculated intranasally with C/OK (donors) and from swine exposed to inoculated swine by direct contact. Numbers of pigs positive by rt-RT-PCR are indicated above or below each time point. (TIFF) [file ppat.1003176.s001.tiff]

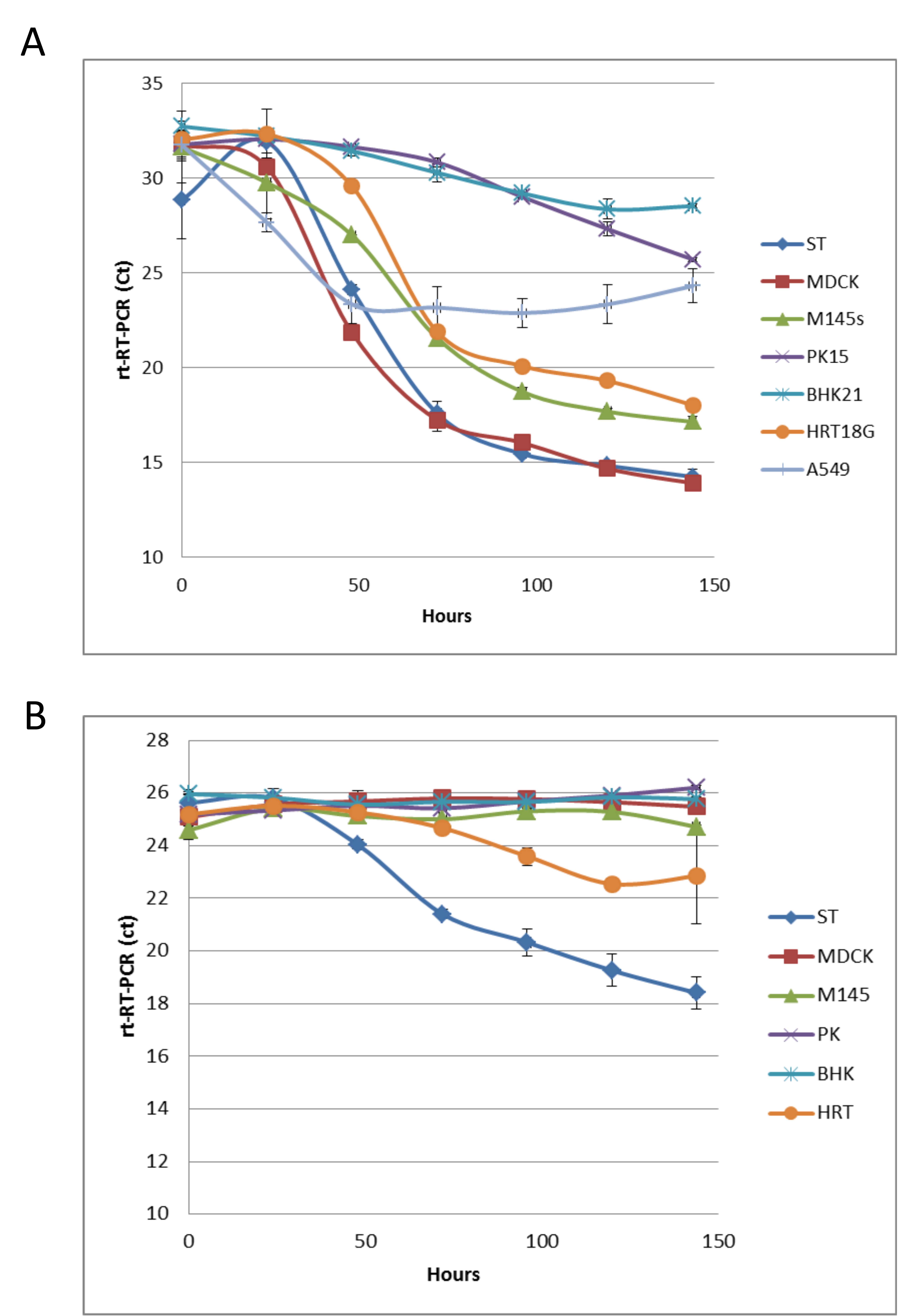

Supplement: Figure S2 — Growth of influenza C/swine/Oklahoma/1334/2011 (A) and influenza C/Taylor/1233/1947 (B) in cell cultures. ST, MDCK, Marc145, PK or -15, BHK-21, HRT-18G and A549 cell lines were inoculated with approximately 1.0–3.0 log10 TCID50/mL of influenza C/OK virus or influenza C/Taylor virus (MOI = 1×10^−5^–1×10^−3^). Virus was titrated at the indicated time points by rt-RT-PCR. Experiments were done in duplicate; values are the mean of duplicate samples and error bars represent the standard deviation. (TIF) [file ppat.1003176.s002.tif]

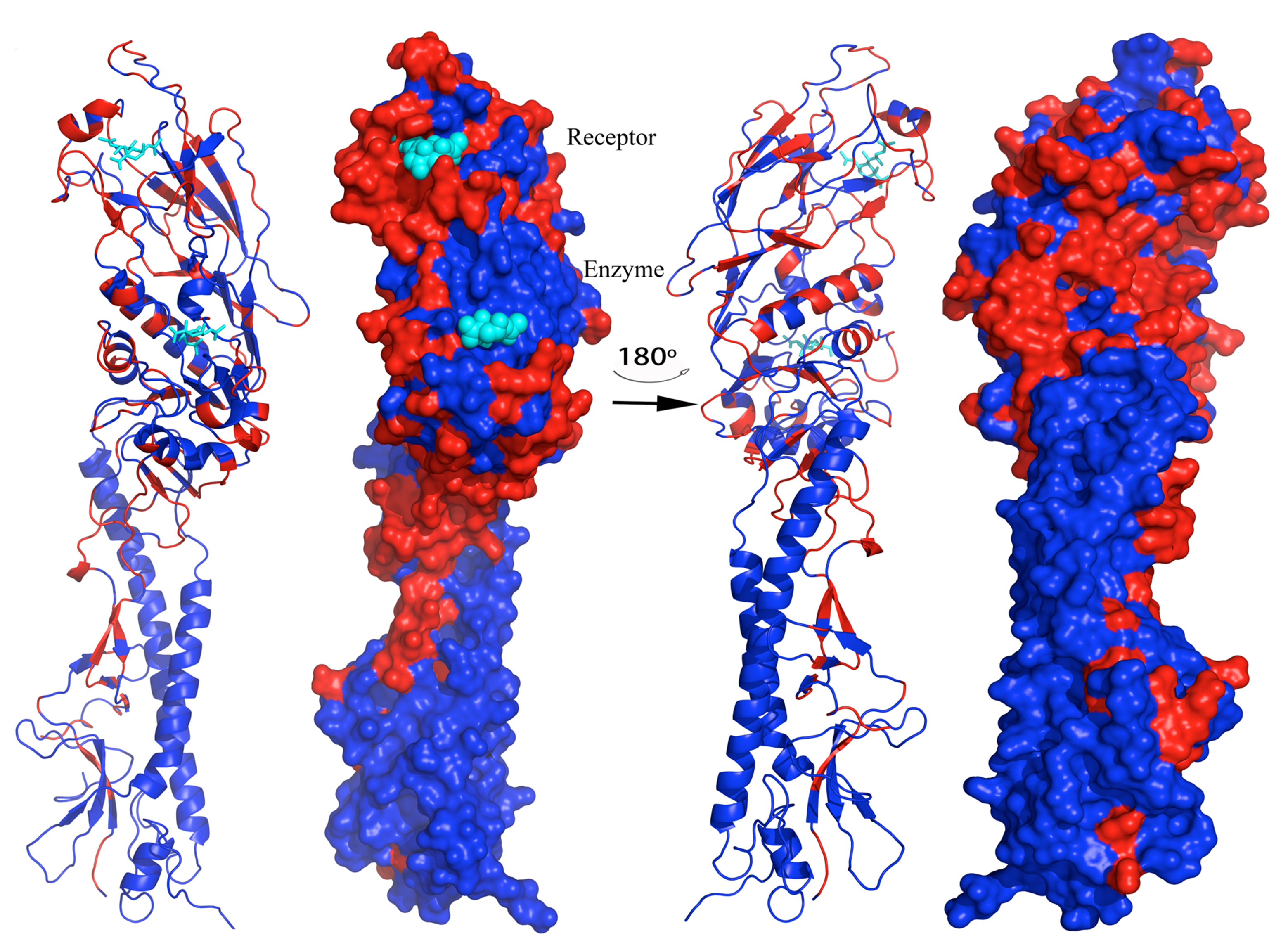

Supplement: Figure S3 — Modeled structure of C/OK HE protein. Cartoon and surface representations of HE structure are shown below. HE was colored blue. Residues that are not identical in C/Johannesburg/1/66 HE were marked red. An analog of 9-O-sialic acid, 9-acetamindo-sialic acid α-methylglycoside (cyan), was manually docked to the binding sites of receptor and esterase domain according to a previous study [36]. (TIFF) [file ppat.1003176.s003.tiff]

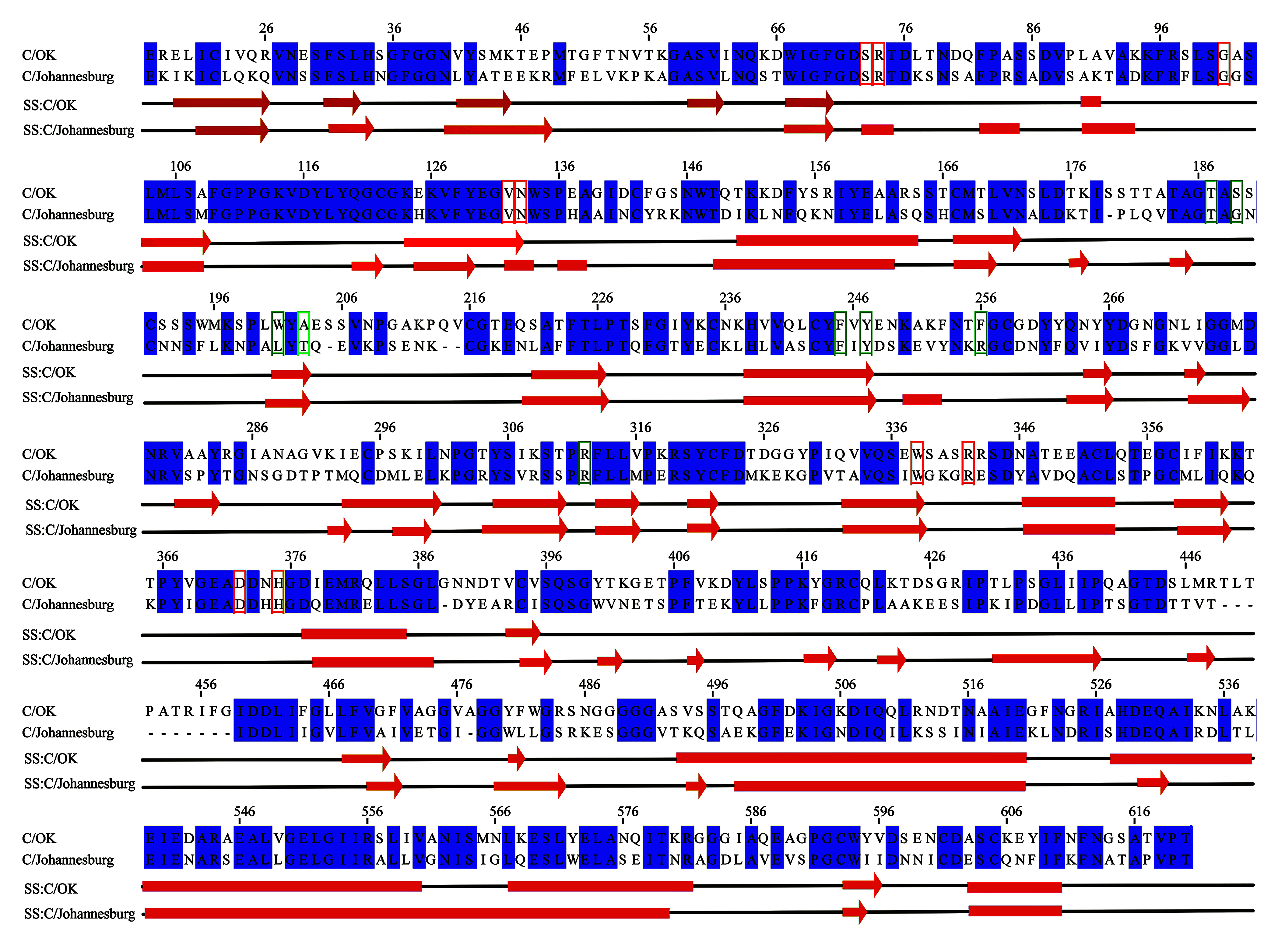

Supplement: Figure S4 — Sequence alignment and secondary structure of HE protein. Sequences were aligned using MUSCLE [57]. Esterase active site residues and receptor binding site residues of human influenza C HE protein are marked with red and blue rectangles, respectively. Secondary structure of C/OK HE was predicted using PSIpred while that of human influenza C HE protein is from PDB structure (1FLC) [58]. Pink rectangles represent α helix, orange arrows represent β strands and black lines are random coils and loops. (TIF) [file ppat.1003176.s004.tif]
