# Supplementary material for: Isolation of a Novel Swine Influenza Virus from Oklahoma in 2011 Which Is Distantly Related to Human Influenza C Viruses
Source: PLoS Pathog. 2013 Feb 7;9(2):e1003176. doi: 10.1371/journal.ppat.1003176 (PMC3567177; doi:10.1371/journal.ppat.1003176)
Supplement: Table S1 — Sequences of the 3′ and 5′ noncoding regions of the genomic segments of C/swine/Oklahoma/1334/2011 (A) and C/JHB/1/66 (B). (DOCX) [file ppat.1003176.s005.docx]

**Table S1. Sequences of the 3’ and 5’ noncoding regions of the genomic segments of C/swine/Oklahoma/1334/2011 and C/JHB/1/66**

A)

| **C/swine/Oklahoma/1334/2011** | | |
| --- | --- | --- |
| **Segment** | **3' end non coding sequence^a^** | **5' end non coding sequence** |
| PB2 | C**CGUAUUCGUC**UC**C**UAC | **AGCAGUAGCAAG**AG**G**A*UUUUUU*CAAUGUGCUUCA |
| PB1 | C**CGUAUUCGUC**UC**C**UAAAAUAUUGUUAC | **AGCAGUAGCAAG**AG**G**A*UUUUU*CUGUUAUUAAACAACGCAAAGCUUA |
| P3 | C**CGUAUUCGUC**CU**C**UAAAUCUUUAC | **AGCAGUAGCAAG**GA**G**A*UUUUU*AACAUUACAAGGCCUUUGGUCA |
| HEF | U**CGUAUUCGUC**CU**C**UAAAAGUUUCUAC | **AGCAGUAGCAAG**GA**G**A*UUUUUU*CUAAGAUUCUA |
| NP | C**CGUAUUCGUC**CU**C**UAAUAAUUCGUUAUAC | **AGCAGUAGCAAG**GA**G**A*UUUUUU*GUUAAAUAAGACAAACCAACAUCUUUAACACCCACUGGGGACUGCAACAGAACCAUCCAAAGAUGAGUUA |
| M | U**CGUAUUCGUC**UC**C**UAUAAAAACUCGCUUAC | **AGCAGUAGCAAG**AG**G**A*UUUUUU*CGCGAUUA |
| NS | U**CGUAUUCGUC**CC**C**ACAUGUUAAAGUUAUAC | **AGCAGUAGCAAG**GG**G***UUUUUU*CA |

B)

| **C/JHB/1/66** | | |
| --- | --- | --- |
| **Segment** | **3' end non coding sequence** | **5' end non coding sequence** |
| PB2 | **UCGUCUUCGUC**UC**C**UAACCUUUAC | **AGCAGUAGCAAG**AG**G**A*UUUUU*A |
| PB1 | **UCGUCUUCGUC**UC**C**UAAUAC | **AGCAGUAGCAAG**AG**G**A*UUUUUU*CAUUUAAUGGAAUAACAAAAAUAUGUGCAAGUAGGAGGAAAGGGUUUAACAGCCCCUCCUCA |
| P3 | **UCGUCUUCGUC**CC**C**UAGGCUUUAC | **AGCAGUAGCAAG**GG**G**A*UUUUUU*CUUAUAAUGAUCA |
| HEF | **UCGUCUUCGUC**CC**C**CAAUUAUUAC | **AGCAGUAGCAAG**GG**G**A*UUUUU*GUUUUUUAUAAAACAGUACAAAAUAUUGACCAACACAUUAUCCAUUUUUCAAAAUUGUCUCAAUCA |
| NP | **UCGUCUUCGUC**CU**C**UAAACCAAAAGUUUUUAC | **AGCAGUAGCAAG**GA**G**A*UUUUU*GAAUUAUAUAUAGCAAUACAACAGUUGAUCAUAAAAUGUGCGAUGAAUUUAAUCUGACUUUAAUUUUCUCCAGGAAUGUUGCUA |
| M | **UCGUCUUCGUC**CC**C**UGAAAAUUUGUUAC | **AGCAGUAGCAAG**GG**G**A*UUUUUU*CAAGGUAAUUA |
| NS | **UCGUCUUCGUC**CC**C**AUGAAAAAGUUUUAC | **AGCAGGAGCAAG**GG**G***UUUUUU*AACUUUGGAAUAACAACUUAAAACAAUUA |

Conserved sequences in bold; start and stop codons underlined; poly U stretch in italic
