# Supplementary material for: Isolation of a Novel Swine Influenza Virus from Oklahoma in 2011 Which Is Distantly Related to Human Influenza C Viruses
Source: PLoS Pathog. 2013 Feb 7;9(2):e1003176. doi: 10.1371/journal.ppat.1003176 (PMC3567177; doi:10.1371/journal.ppat.1003176)
Supplement: Table S2 — Cross-reactivity of antibodies to influenza A, B and C viruses and C/swine/Oklahoma/1334/2011 virus as measured by HI assay using turkey red blood cells. (DOCX) [file ppat.1003176.s006.docx]

**Table S2.**  **Cross-reactivity of antibodies to influenza A, B and C viruses and C/swine/Oklahoma/1334/2011 virus as measured by HI assay using turkey red blood cells**

| **Virus** | **A/CA** | **A/NC** | **A/MN** | **B/Florida** | **C/OK** | **C/Taylor** |
| --- | --- | --- | --- | --- | --- | --- |
| A/CA/04/2009(H1N1) | **160** | <10 | <10 | <10 | <10 | <10 |
| A/swine/NC/6300-1/2010(H1N2) | <10 | **320** | <10 | <10 | <10 | <10 |
| A/swine/MN/3793/2008(H1N1) | <10 | <10 | **320** | <10 | <10 | <10 |
| B/Florida/2006 | <10 | <10 | <10 | **160** | <10 | <10 |
| C/swine/OK/1334/2011 | <10 | <10 | <10 | <10 | **≥1280** | <10 |
| C/Taylor/1233/1947 | <10 | <10 | <10 | <10 | <10 | **320** |
