# Supplementary material for: Isolation of a Novel Swine Influenza Virus from Oklahoma in 2011 Which Is Distantly Related to Human Influenza C Viruses
Source: PLoS Pathog. 2013 Feb 7;9(2):e1003176. doi: 10.1371/journal.ppat.1003176 (PMC3567177; doi:10.1371/journal.ppat.1003176)
Supplement: Text S1 — Supplementary Materials and Methods. (DOC) [file ppat.1003176.s007.doc]

**Supplementary Materials and Methods for**

**Isolation of a novel swine influenza virus from Oklahoma in 2011 which is distantly related to influenza C**

Ben M. Hause^1,2,*^, Mariette Ducatez^3^, Emily A. Collin^1^, Zhiguang Ran^2,4^, Runxia Liu^2,4^, Zizhang Sheng^5^, Anibal Armien^6^, Bryan Kaplan^3^, Suvobrata Chakravarty^5^, Adam D. Hoppe^5^, Richard J. Webby^3^, Randy R. Simonson^1^, Feng Li^2,4*^

* To whom correspondence should be addressed.

Email: [bhause@newportlabs.com](mailto:bhause@newportlabs.com) (B.M.H); [feng.li@sdstate.edu](mailto:feng.li@sdstate.edu) (F.L)

**Materials and Methods**

***Viruses***

Influenza B/Florida/04/2006 and its ferret antiserum were a gift from Drs. Xiyan Xu and Ruben Donis (CDC), influenza C/Taylor/1233/47 and its chicken antiserum were provided by BEI Resources (NIAID), and C/Yamagata/10/1981 was kindly provided by Peter Palese.

***Electron Microscopy***

Negative-contrast electron microscopy was performed for detection of virus particles. Infected ST cells were placed in 1 ml of double-distilled water and centrifuged at 2,900 x *g* for 10 min. The supernatant was re-centrifuged in an airfuge (Beckman Coulter) at 30 PSI for 10 min. The supernatant was discarded and the pellet was suspended in 10 µl of double-distilled water. The suspension was placed on formvar-coated copper grids (Electron Microscopy Services) and stained with 1% phosphotungstic acid for 1 min.

Thin-section electron microscopy was performed to detect virions budding from infected cells. Infected ST cells were fixed in 0.166 M cacodylate-buffered 3% glutaraldehyde with 1% tannic acid solution, followed by treatment in 1% osmium tetroxide. Ultrathin sections (80 nm) were stained with uranyl acetate and lead citrate. All electron microscopy work was conducted at the University of Minnesota Veterinary Diagnostic Laboratory.

***Genetic Analysis***

Virus (300 ml) was harvested from ST cell culture by two freeze/thaw cycles. Cell supernatants were centrifuged at 5000 x *g* for 10 min to remove cell debris and were then filtered through a 0.2 µm bottle-top filter. Virus was pelleted by centrifugation at 100,000 x *g* for 3 h, and the viral pellet was resuspended in 1 mL of phosphate buffered saline (PBS) and digested with DNase and RNase to removed cellular genetic materials. The digest was then applied to a 25% sucrose cushion and centrifuged for 3 h at 100,000 x *g*. The virus pellet was resuspended in 700 µl AVL (Qiagen Viral RNA Isolation Kit) and viral RNA was isolated by following the manufacturer’s instructions. 10 µL of viral RNA was reverse-transcribed by using random primers and the GoScript Reverse Transcription Kit (Promega) according to the manufacturer’s instructions. After reverse transcription, 2 µl of Klenow fragment was added to the reaction, which was then incubated at 37°C for 1 h. A viral cDNA library was prepared by using the NEBNext Fast DNA Fragmentation and Library Prep Set 4 kit (New England Biolabs) according to the manufacturer’s instructions. DNA sequencing templates were prepared by using the Ion Xpress Template Kit version 2.0 (Life Technologies) and sequenced in an Ion Torrent Personal Genome Machine (Life Technologies). Contigs were assembled *de novo* by using SeqMan NGen software (DNAStar). Contigs encoding proteins with homology to influenza C proteins were identified by BlastP analysis. The genome sequence of C/OK was submitted to Genbank under accession no. JQ922305-JQ922311. Phylogenetic analyses were performed by using Mega 5 software [51]. Evolutionary analyses were conducted by using the Maximum Likelihood algorithm, and the tree topology was verified by performing 1000 bootstrap replicates.

***Development of a real-time reverse transcriptase PCR method to detect C/OK***

As PB1 is the most conserved influenza virus gene, the PB1 sequence was used to design primers and a Taqman probe for detection of C/OK. Primers “C/OK Forw” (5'-GCT GTT TGC AAG TTG ATG GG-3') and “C/OK Rev” (5'-TGA AAG CAG GTA ACT CCA AGG-3') were used with probe “C/OK Probe” (5'-TTC AGG CAA GCA CCC GTA GGA TT-3'), which contained the Cy5 fluorophore. Viral RNA was extracted by using the MagMAX-96 viral RNA isolation kit (Life Technologies) according to the manufacturer’s instructions. rt-RT-PCR was performed by using QIAGEN Quantitect RT-PCR with the C/OK primers and probe. For analytical purposes, negative samples were assigned a Ct value of 37.1, which corresponds to the detection limit of the method (approximately 10 TCID_50_/mL). Method specificity was assessed by using influenza A, B, and C reference viruses, and no cross-reaction was observed. A standard curve was generated by serial dilution of ST cell harvests containing 6.2 log_10_ TCID_50_/mL of C/OK, as determined by titration on ST cells.

***Serology***

Human sera were treated with receptor-destroying enzyme (Denka Seiken Co., Tokyo, Japan) overnight at 37°C, heat-inactivated at 56°C for 30 min, diluted 1:10 with PBS, and tested by hemagglutination inhibition (HI) assay with 0.5% packed chicken red blood cells (cRBCs) as described in the WHO Manual on Animal Influenza Diagnosis and Surveillance [52].

***Assessment of virus pathogenicity and transmission in ferrets***

The pathogenicity and transmission of the virus was tested in 3- to 4 month-old male ferrets (*Mustela putorius furo*) obtained from Marshall Farms (North Rose, NY). All ferrets were seronegative for circulating influenza A H1N1 and H3N2 and influenza B viruses. Ferrets to be inoculated (donor ferrets) were initially housed separately from contact ferrets. The three donors were inoculated intranasally under light isoflurane anesthesia with 10^6^ TCID_50_ of swine/Oklahoma/1334/2011 virus in 1 ml of sterile PBS. Two additional ferrets were similarly inoculated and were housed separately for virus titration and histopathology in organs. At 23 h p.i., each of the three remaining donor ferrets was housed in a cage with one naïve direct-contact ferret (n=3). An additional ferret (n=3) was placed in an adjacent cage separated from the donor's cage by a two layers of wire mesh (~5 cm apart) that prevented physical contact but allowed the passage of respiratory droplets. Clinical signs of infection, relative inactivity index [53], weight, and temperature were recorded on days 0, 3, 5, 7, and 10 p.i.. Body temperature was measured by subcutaneous implantable temperature transponders (Bio Medic Data Systems Inc, Seaford, DE) implanted one week prior to infection.

To monitor virus shedding, nasal washes were collected from ferrets 3, 5, 7, and 10 days p.i. Virus was titrated in ST cells as log_10_ TCID_50_/ml. Two donor animals were euthanized under isoflurane anesthesia by intracardiac injection of Euthanasia V solution 5 dpi, and tissue samples (~0.5 g each) were collected from lungs (4 to 5 lobes tested separately), nasal turbinate, trachea, spleen, liver, and small intestine. Samples were homogenized in 1 ml of culture medium with antibiotics, and virus was titrated (log_10_ TCID_50_ per gram of tissue) in ST cells. Tissues (lung, nasal turbinate, trachea, spleen, liver, and small intestine) were also collected for histopathologic analysis. They were fixed in 10% neutral-buffered formalin and embedded in paraffin. Sections 5 µm thick were stained with hematoxylin and eosin and studied by light microscopy. Serum samples were collected from ferrets 3 weeks after inoculation and tested by hemagglutination inhibition assay.

Animal experiments were performed in biosafety level 2+ facilities at St. Jude Children’s Research Hospital (Memphis, TN). All animal studies were approved by the St. Jude Animal Care and Use Committee and were conducted according to applicable laws and guidelines.

***Assessment of virus pathogenicity and transmission in swine***

Swine challenge studies were performed at Newport Laboratories under biosafety level 2 conditions. Twenty-eight swine approximately 10 weeks of age were obtained from a commercial high-health herd. The swine were negative for influenza A antibodies as determined by commercial ELISA (FlockChek Avian Influenza MultiS-Screen Antibody Test Kit; Idexx Laboratories, Inc.) and influenza C antibodies as determined by the hemagglutination inhibition assay using C/OK. Nasal swabs were also determined to be negative for influenza A and C/OK by rt-RT-PCR before the animals were used. Eleven swine were placed in a single room and inoculated intranasally with 6.0 log_10_ TCID_50_ of C/OK. On day 1 p.i., 11 naïve direct-contact swine were introduced into the room. Six swine housed in an adjacent clean room were mock-inoculated with DMEM to serve as negative controls. Temperatures were recorded and nasal swabs were collected on days 0, 2, 3, 6, 8, and 10 p.i. Six inoculated swine and three mock-inoculated swine were euthanized on day 7 p.i. and lung specimens were fixed in 10% neutral buffered formalin. At the Iowa State Veterinary Diagnostic Laboratory, the tissues were stained with hematoxylin and eosin and studied by light microscopy. The remaining swine were euthanized on day 14 p.i. Nasal swabs and lung tissue were analyzed by rt-RT-PCR as described above.
